# The Effect of Psychological *Suzhi* on Problem Behaviors in Chinese Adolescents: The Mediating Role of Subjective Social Status and Self-esteem

**DOI:** 10.3389/fpsyg.2017.01490

**Published:** 2017-08-31

**Authors:** Guangzeng Liu, Dajun Zhang, Yangu Pan, Yuanxiao Ma, Xingyue Lu

**Affiliations:** ^1^Faculty of Psychology, Southwest University Beibei, China; ^2^Research Institute of Social Development, Southwestern University of Finance and Economics Chengdu, China

**Keywords:** psychological *suzhi*, problem behaviors, subjective social status, self-esteem, adolescents

## Abstract

In this study, we examined subjective social status (SSS) and self-esteem as potential mediators between the association of psychological *suzhi* and problem behaviors in a sample of 1271 Chinese adolescents (44.5% male, grades 7–12). The results showed that SSS and self-esteem were fully mediating the relationship between psychological *suzhi* and problem behaviors. Moreover, the indirect effect was stronger via self-esteem than via SSS. These findings perhaps provide insight into the preliminary effect that SSS and self-esteem underlie psychological *suzhi*’s effect on adolescents’ problem behaviors, and also are important in helping school-teachers and administrators to develop a better understanding of problem behaviors in their schools as a pre-requisite to the development of more effective behaviors management practices from the perspective of psychological *suzhi.* Implications and limitations in the present study have also been discussed.

## Introduction

For adolescents, problem behaviors refer to the harmful behaviors to their life, physical, and mental health (e.g., fighting, smoking, and alcohol use; DiClemente et al., 1996; Zhu et al., 2016). Problem behaviors have a great impact on the physical and mental health of adolescents (Karaman, 2013), for example, the failure model theory suggested that the problem behaviors precede depression, which in turn lead to depression (Capaldi, 1992; Capaldi and Stoolmiller, 1999). Therefore, in order to provide theoretical guidance for reducing and solving problem behaviors among adolescents, research needs to examine factors predicting and underlying problem behaviors. Although extensive research had examined problem behaviors, but no research had examined if psychological *suzhi* affects problem behaviors in adolescents during middle school stage, or aimed at identifying a mechanism underlying such an effect.

Psychological *suzhi* is a native academic conception first proposed by Chinese scholars within the context of quality-oriented education (Zhang et al., 2000; Zhang, 2003, 2012). As a Chinese-originated conception, there is no corresponding conception in the Western psychology academia. In Chinese psychology domain, Psychological *suzhi* had also been translated as *mental quality* or *psychological quality.* However, the word *quality* cannot cover the whole meaning of *suzhi*, and this is the reason why it is coined psychological *suzhi*. Although psychological *suzhi* is originated in China, it shares common connotations with traditional conceptions in western psychology. For example, psychological *suzhi* focuses on individual’s positive and initiative psychological development and emphasizes individual’s adaptation toward environment, which is consistent with the proposal of positive psychology in western psychology (Zhang et al., 2011; Wang et al., 2015). Psychological *suzhi* is a concept of Chinese positive psychology from a certain point of view, which has gained acceptance and recognition in Western academia and been collected in the Handbook of Positive Psychology in Schools (2nd edition), an international authoritative reference book (Furlong et al., 2014).

Psychological *suzhi* is a kind of comprehensive quality, it is defined as a mental quality characterized by being steady (for a certain period of time remain unchanged), essential (fundamental, throughout the development of the individual), and implicit (an intangible mental activity that cannot be directly observed, measured, or recorded by the outside), which has a derivative function related with individuals’ developmental, adaptive, and creative behaviors and a multi-level self-organized system that involves steady implicit mental qualities (content elements, include cognitive quality and individuality) and explicit adaptive (functional value, include adaptability) behaviors (Zhang et al., 2000, 2011; Zhang, 2003, 2012). Moreover, empirical research has supported the theoretical consideration that psychological *suzhi* has three dimensions: cognitive quality, individuality, and adaptability. As the most basic component of psychological *sushi*, cognitive quality is directly involved in individuals’ cognition of objects. Individuality is not directly involved in individuals’ cognition of objects, but reflected in individuals’ actions toward objects. Individuality is related to the concept of personality and has a motivating and moderating function during cognition, which is the core component of psychological *suzhi*. Adaptability is a reliable indicator of the other two dimensions’ activity in various social environments and governs individuals’ ability to achieve consistence between themselves and the environment by changing themselves or the environment during socialization. Adaptability is also a comprehensive reflection of cognitive quality and individuality in individual’s adaptation–development–creative behavior (or explicit behavioral habit). The three basic dimensions of psychological *suzhi* are interrelated and differentiated, which constitute the core elements of psychological *suzhi* (Zhang et al., 2000, 2011; Zhang, 2003).

Psychological *suzhi* has certain stability, it can also be changed to a certain extent under certain conditions and after a certain period of time, especially for children and adolescents, in other words, psychological *suzhi* is not only a stable mental quality, but also plastic, whereas mental health is a favorable and positive psychological state according to theoretical models of psychological *suzhi*’s relationship with mental health (Zhang et al., 2011; Zhang and Wang, 2012). Psychological *suzhi* directly predicts individuals’ mental health, and mental health indicates sound psychological *sushi*. The relationship between psychological *suzhi* and mental health is just like the relationship between “essence” and “surface,” that is to say, the psychological *suzhi* of adolescents is the core of their psychological structure and the basis of their mental activity (play a dominant role), and mental health is the state layer of their psychological structure (surface or explicit layer), which reflects the state of psychological *suzhi* (surface) (Zhang et al., 2011; Zhang and Wang, 2012). Generally, individuals with high level of psychological *suzhi* seldom have mental health problems. In contrast, individuals with low level of psychological *suzhi* are more likely to suffer from mental disturbances. Mental health not only has its external state or symptoms, but also its intrinsic and endogenous factors (Wang and Zhang, 2011). The essence of mental health is good psychological *suzhi*, good adaptation, and its behaviors are the external manifestations of mental health (Shen and Ma, 2004). The key to mental health education is to improve the students’ psychological *suzhi*. The cultivation of adolescents’ perfect psychological *suzhi* is one of the basic ways to solve the psychological problems of adolescents (Lin, 2000; Zhang, 2008). For example, psychological *suzhi* significantly negatively predicted depression in children and adolescents from previous empirical researches (Hu and Zhang, 2015; Su and Zhang, 2015).

Problem behaviors is defined as the abnormal behaviors of individuals that hinder their social adaptation by Chinese scholars (Lin et al., 2005), which matches the function of psychological *suzhi* according to the concept and structure of psychological quality (Zhang et al., 2000, 2011; Zhang, 2003). Good adaptation and its behaviors (e.g., adapt to and integrate into the social environment effectively) are the external manifestations of mental health (Shen and Ma, 2004), which means that problem behaviors are the external manifestations of mental health. Besides, research had proved that psychological *suzhi* significantly negatively predicted problem behaviors in children (Wu et al., 2015). So we predicted that psychological *suzhi* would be negatively correlated with adolescents’ problem behaviors. However, there was no research has examined if psychological *suzhi* affects problem behaviors in adolescents. Therefore, our first purpose of this study was to test if psychological *suzhi* affects problem behaviors in adolescents. We hypothesized that psychological *suzhi*’s relationship with problem behaviors and mental health would be similar. Psychological *suzhi* is likely to predict reduced problem behaviors in adolescents, then the mature interventions (e.g., the level of adolescents’ psychological *suzhi* can be raised through the intervention of parent–child relationship) that could help children and adolescents to cultivate and improve their psychological *suzhi* would be used to reduce and solve adolescent’s problem behaviors (Zhang et al., 2011, 2014; Zhang, 2012). Examination of psychological *suzhi*’s association with problem behaviors may extend the understanding of problem behaviors’ causes and inform new interventions targeting problem behaviors.

The second purpose of this study was to identify a mechanism underlying the effect of psychological *suzhi* which affects problem behaviors in adolescents. Researchers have been doing a lot of studies on the influencing factors of problem behaviors. Among these factors, subjective social status (SSS) may be an important factor of concern. Subjective social status is defined as the individual’s subjective perception and belief of his or her social class (Singh-Manoux et al., 2003). In some previous research, adolescents with a high social status would prefer to exclude others and had more aggression problem behaviors (Cillessen and Mayeux, 2004; Ahn et al., 2010). However, compared to social status, SSS may more accurately capture the consequential aspects of social status (Goodman et al., 2003). In addition, SSS was closely related to the physical and mental health of people of all ages, and the scores of anxiety, depression, various somatic diseases, and dangerous behaviors were lower in high level SSS individuals (Hu et al., 2012), psychological *suzhi* could positively and significantly predict adolescents’ SSS (Liu et al., 2017). Therefore, psychological *suzhi* may be correlated with adolescents’ SSS, and we predicted that SSS importantly mediates the relationship between psychological *suzhi* and adolescents’ problem behaviors.

Self-esteem is another important factor of concern that influences problem behaviors. Self-esteem refers to the individual’s positive evaluation of self-worth and related experience gained in the process of social comparison, which is an affirmative or negative evaluation of oneself and one’s own possession, indicating the extent to which the individual believes that he or she is important, capable, and valuable (Coopersmith, 1967). According to Maslow’s hierarchy of needs theory, self-esteem embodies the individual’s need for respect and belongs to the individual’s advanced needs. Moreover, when the individual’s respect needs to be met, it will make people full of confidence in themselves, to experience the meaning and value of their own lives (Huitt, 2004). On the other hand, adolescence is the “mighty storm” of life developmental period. With the rapid changes in physiology, adolescents’ psychology has undergone tremendous changes. They desire to get respect from parents, teachers, and classmates, and are also eager to get their approval according to the psychological development of adolescents. There is no doubt that high level of self-esteem contributes to the physical and mental health of adolescents (Orth and Robins, 2014). Terror management theory and its anxiety-buffer hypothesis suggest that self-esteem is an “anxiety-buffer,” which the self-regulation mechanism of self-esteem provided the elastic space could ease anxiety, individuals with high level self-esteem are less likely to develop anxious moods and are less prone to anxiety-related behaviors, while low level self-esteem individuals are more prone to anxiety, anxiety-related behaviors, and social adjustment difficulties (Greenberg et al., 1992; Harmon-Jones et al., 1997; Lei and Zhang, 2003). Self-esteem is the core element of mental health (Yang and Zhang, 2003), it could effectively predict adolescents’ well-being and depression (Furnham and Cheng, 2000; Cheng and Furnham, 2003). High level of self-esteem was found to be associated with reduced mental health symptoms in adolescents (Qian and Xiao, 1998). Psychological *suzhi* significantly positively predicted adolescents’ self-esteem (Liu et al., 2016). Therefore, we predicted that self-esteem importantly mediates the relationship between psychological *suzhi* and adolescents’ problem behaviors.

**FIGURE 1:**
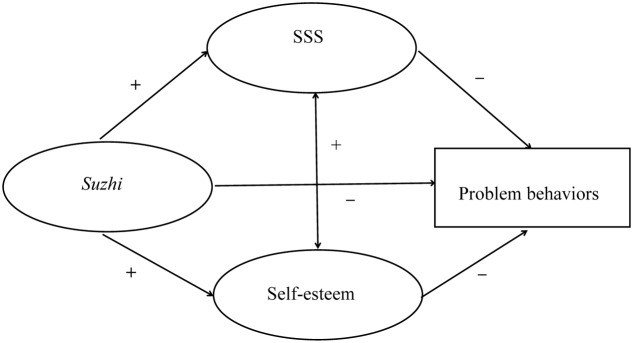
Model of the hypothesized mediator role of SSS and self-esteem in the relationship 164 between psychological *suzhi* and problem behaviors.

Based on the literature review, the current study first attempted to explore the relationships between psychological *suzhi*, SSS, self-esteem, and problem behaviors in Chinese adolescents, and subsequently attempted to examine the psychological mechanisms that account for these associations. Thus, we hypothesized that:

H1: psychological *suzhi* would be positively associated with adolescents’ SSS and self-esteem.
H2: SSS and self-esteem would be negatively associated with adolescents’ problem behaviors.
H3: SSS and self-esteem would mediate the association between psychological *suzhi* and problem behaviors.
  A detailed model of the hypothesized mediator role of SSS and self-esteem in the relationship between psychological *suzhi* and problem behaviors is presented in **Figure 1**.

## Materials and Methods

### Participants

The Research Ethics Committee of Southwest University approved the study. The participants were 1271 adolescents recruited from three middle and high schools that include middle and high school students in Chongqing, China. Participants were aged 15.13 ± 1.84 years ranged from 11 to 19 years old (only 2 participants were 11 years old; 85 participants were 12 years old; 1161 participants were 13–18 years old; and 23 participants were 19 years old). Among them, 215 were in seventh grade, 209 were in eighth grade, 222 were in ninth grade, 209 were in tenth grade, 221 were in eleventh grade, and 195 were in twelfth grade. There were 575 boys and 696 girls. Participants were all of Han ethnicity.

We obtained both written and informed consent from all participants and their parents. Participants completed a battery of paper-based questionnaires, including the Psychological *Suzhi* Questionnaire for Middle School Students (PSMQ), the Subjective Social Status Questionnaire (SSSQC), the Rosenberg Self-esteem Scale (RSES), and the Chinese version of Strength and Difficulties Questionnaire (Ch-SDQ). Participants completed the battery of questionnaires in their respective classrooms in 30 min.

### Measures

#### Psychological *Suzhi*

We examined psychological *suzhi* using the PSMQ (Hu et al., 2017). This questionnaire was developed and validated based on the bi-factor model (Reise et al., 2013; Vecchione et al., 2014), each item or dimension is independent and contributes to overall psychological *suzhi*. The questionnaire includes 24 items (each dimension includes eight items) examining the following dimensions of psychological *suzhi*: cognitive quality, individuality, and adaptability (example items from each dimension: *I am good at linking old and new knowledge to study*, *I usually do my own thing by myself*, and *I often can effectively resolve the embarrassment*, respectively), and it is suitable to use for adolescents in the Chinese school environment. Participants indicated their responses to each item on a 5-point Likert scale from 1 = *totally disagree* to 5 = *totally agree*. Scores range from 24 to 120, higher scores indicate greater psychological *suzhi*. Psychological *Suzhi* Questionnaire for Middle School Students has good internal consistency reliability for the total scale (α = 0.91; Hu et al., 2017). In the present study, the Cronbach’s alpha of total scale was 0.91, and the total score was used.

#### Subjective Social Status

We examined SSS using the SSSQC for College Students (Cheng et al., 2015). The SSSQC is a single-factor construct that contains seven items examining the following topics: academic achievement, family conditions, popularity, social practice ability, talent level, emotional state, and image temperament. The SSSQC uses a 10-rung “ladder” to measure SSS: for each item, participants indicate their position on the ladder. Higher positions indicate greater SSS. For scoring, each rung of the ladder was assigned a numerical value corresponding to its height (i.e., the highest and lowest rungs were coded as 10 and 1, respectively). In the present study, the SSSQC showed that good internal consistency reliability was α = 0.87, and the total score was used.

#### Self-esteem

We examined self-esteem using the RSES (Rosenberg, 1986). The RSES contains 10 items (example item: *I have a positive attitude toward myself*, respectively). Participants indicated their responses to each item on a 4-point rating scale from 1 = not at all to 4 = very much. Scores range from 10 to 40, higher scores indicate greater self-esteem. The RSES has been widely used to test self-esteem and has shown good validity and reliability (Wang et al., 1999). However, due to the cultural differences and controversies between the Chinese and Western cultures in item 8 (*I hope I can win more respect for myself*), Chinese subjects tend to choose very much, this study adopts the practice of Tian (2006), and we deleted it. In the present study, the RSES’ internal consistency reliability was α = 0.81 for the total scale included nine items, and the total score was used.

#### Problem Behaviors

We examined problem behaviors using the Ch-SDQ (Dai, 2010) translated and revised from Goodman (2001). The SDQ was used to test problem behaviors contains 20 items examining the following four dimensions of problem behaviors: emotional symptoms, conduct problems, hyperactivity, and peer problems with five items for each (example items from each dimension: *I often have a headache*, *stomachache or bad health*, *I usually do things according to the orders*, *I’ll think about it before I do something*, and *I have one or a few good friends*, respectively). Participants indicated their responses to each item on a 3-point rating scale from 0 = *not true* to 2 = *certainly true*. The self-reported Chinese SDQ has proven reliable and valid in previous studies in Chinese adolescents (Zhao et al., 2013). Higher scores on the SDQ indicate more problem behaviors. In the present study, the SDQ’s internal consistency reliability was 0.70, and the total score was used.

### Data Analyses

Descriptive analysis was conducted with the variables of interest for the total sample. Then, SEM was carried out to test if SSS or self-esteem mediates the relationship between psychological *suzhi* and problem behaviors (**Figure 1**). Mplus 7.0 was used to evaluate the hypothetical model’s data fit (Muthén and Muthén, 1998–2012). Missing data were handled using the full information maximum likelihood (FIML) procedure. SEM was performed using the robust maximum likelihood (MLR) estimator to account for identified data non-normality. Indirect effects were tested using bootstrapping procedures (Preacher and Hayes, 2008). In order to control for possible inflated measurement errors resulting from multiple items examining the latent variables of psychological *suzhi*, SSS and self-esteem in the present study, we adapted random assignment (Matsunaga, 2008) to create separate item parcels for variables of SSS and self-esteem that because they are single-dimensional variables, and adapted internal-consistency approach (Bandalos, 2002) to create separate item parcels for psychological *suzhi* according to its multi-dimensional structure. The number of items in each parcel was roughly equal; therefore, items’ total scores were used to evaluate the hypothetical model’s overall data fit.

## Results

### Descriptive Statistics of the Variables of Psychological *Suzhi*, SSS, Self-esteem, and Problem Behaviors

**Table 1** summarizes the means and standard deviations, and the correlations between *suzhi*, SSS, self-esteem, and problem behaviors.

**Table 1:** Descriptive statistics and bivariate correlations between study variables (*N* = 1271).

Variables	1	2	3	4	5	6
(1) Psychological *suzhi*	–					
(2) SSS	0.44^∗∗^	–				
(3) Self-esteem	0.46^∗∗^	0.42^∗∗^	–			
(4) Problem behaviors	–0.32^∗∗^	–0.33^∗∗^	–0.50^∗∗^	–		
(5) Gender	–0.02	–0.11^∗∗^	–0.06^∗^	0.03	–	
(6) Age	–0.08^∗∗^	–0.11^∗∗^	–0.03	–0.02	0.04	–
Range	24–120	7–70	9–36	0–40	1 = boy 2 = girl	11–19
Skewness	–0.10	–0.13	–0.10	0.50	–0.19	0.07
Kurtosis	0.44	0.09	–0.08	–0.06	–1.97	–0.98
*M*	85.86	40.30	25.71	12.76	1.55	15.13
*SD*	13.00	10.79	4.35	5.03	0.50	1.84


*SSS, subjective social status, ^∗∗^*p* < 0.01, and ^∗^*p* < 0.05.*

### Examining Subjective Social Status and Self-esteem As Potential Mediators of the Link between Psychological *Suzhi* and Problem Behaviors in Chinese Adolescents

**FIGURE 2:**
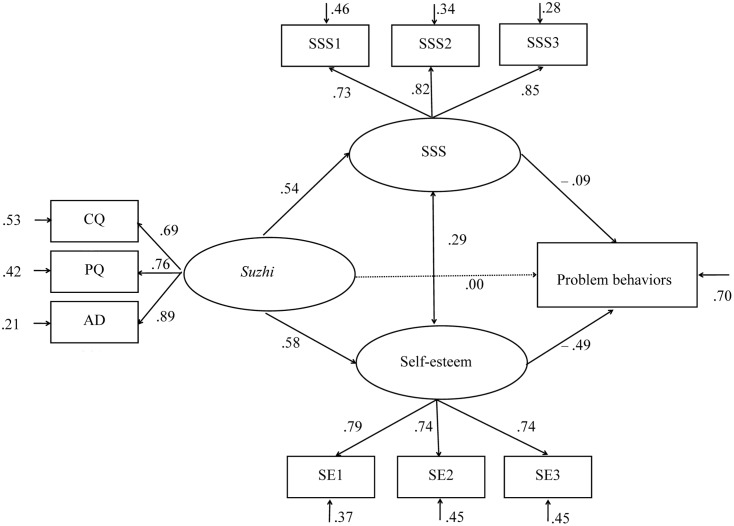
Structural equation model with standardized parameters estimates: psychological *suzhi* and problem behaviors. CQ, cognitive quality; PQ, personality quality; AD, adaptability; SSS, subjective social status, SSS1–SSS3, three parcels of subjective social status; and SE1–SE3, three parcels of self-esteem.

The model examined the associations between psychological *suzhi*, SSS, self-esteem, and problem behaviors (see **Figure 2**). Results showed good data fit, χ^2^_(28, *N* = 1271)_ = 146.446 (*p* < 0.001); CFI = 0.976, TLI = 0.961; RMSEA = 0.058 (90% CI = 0.049, 0.067); and SRMR = 0.034 (Hu and Bentler, 1999). Tests of the indirect effects indicated that SSS and self-esteem fully mediated between psychological *suzhi* and problem behaviors [β = –0.327, SE = 0.033, *p* < 0.001, 95% CI = (–0.389, –0.265)], including specific indirect effects of SSS [β = –0.047, SE = 0.021, *p* < 0.05, 95% CI = (–0.087, –0.007)] and self-esteem [β = –0.280, SE = 0.030, *p* < 0.001, 95% CI = (–0.337, –0.223); **Table 2**]. We subsequently used *model constraint* command of Mplus to create auxiliary variables and used bootstrapping in order to compare the mediation effects (Preacher and Hayes, 2008; Muthén and Muthén, 2010). Psychological *suzhi*’s indirect correlation with problem behaviors was significantly stronger via self-esteem than via SSS [β = –1.172, SE = 0.201, *p* < 0.001, 95% CI = (–1.574, –0.792)].

**Table 2:** Standardized indirect effects from psychological *suzhi* to problem behaviors.

Indirect effect	β (standardized indirect effect)	*SE*	*p*	95% CI (standardized indirect effect)
From psychological *suzhi* to problem behaviors via SSS and self-esteem	–0.327	0.033	<0.001	–0.389, –0.265
Via SSS	(0.54) × (–0.09) = –0.047	0.021	0.027	–0.087, –0.007
Via self-esteem	(0.58) × (–0.49) = –0.280	0.030	<0.001	–0.337, –0.223


*SSS, subjective social status.*

## Discussion

As expected, psychological *suzhi*, SSS, self-esteem, and problem behaviors had significant relationships with each other. Psychological *suzhi* was significantly positively correlated with adolescents’ SSS and self-esteem, which in turn were negatively correlated with adolescents’ problem behaviors. Moreover, we found that SSS and self-esteem fully mediated psychological *suzhi*’s effect on problem behaviors, and the indirect effect was significant stronger via self-esteem than via SSS.

Consistent with prior research, psychological *suzhi* was negatively associated with adolescents’ SSS (Liu et al., 2017). Subjective social status can accurately capture more sensitive aspects of social status than objective indicators and had a greater impact on individuals’ health (Wilkinson, 1999; Goodman et al., 2003). For example, SSS could predict individuals’ well-being and mental health well (Euteneuer, 2014; Haught et al., 2015). Additionally, psychological *suzhi* significantly positively predicted adolescents’ self-esteem was also consistent with prior research (Liu et al., 2016). Self-esteem is an important factor in the promotion of individuals’ physical and mental health (Orth and Robins, 2014). Therefore, as the core element of mental health (Yang and Zhang, 2003), psychological *suzhi* could effectively predict adolescents’ well-being and depression (Furnham and Cheng, 2000; Cheng and Furnham, 2003). The results showed that psychological *suzhi* was significantly positively correlated with adolescents’ SSS and self-esteem, which supports our Hypothesis 1. Both of SSS and self-esteem were positive predictor or core element of mental health that could be significantly positively predicted by psychological *suzhi*, which support and verify the models of *suzhi*’s relationship with mental health (Zhang et al., 2011; Zhang and Wang, 2012). This result indicates us that improving adolescents’ psychological *suzhi* and thereby improving their SSS and self-esteem may promote adolescents’ development of good mental health. Moreover, the current results are preliminary and should inform future longitudinal or experimental studies.

Subjective social status and self-esteem were significantly negatively correlated with adolescents’ problem behaviors, which supports Hypothesis 2. Problem behaviors are generally divided into externalizing problem behaviors (such as aggression and alcohol abuse) and internalizing problem behaviors (such as anxiety and depression) (Achenbach, 1991; Barber et al., 1994). Subjective social status’ significant positive prediction of adolescents’ problem behaviors supports Hu et al. (2012) that higher SSS was closely related to individual’s lower anxiety and depression. Similarly, self-esteem’ significant negative prediction of adolescents’ problem behaviors supports terror management theory and its anxiety-buffer hypothesis. A possible explanation for this result is that as an “anxiety-buffer,” individuals with high level self-esteem are less likely to develop anxious moods and are less prone to anxiety-related behaviors, while low level self-esteem individuals are more prone to anxiety, anxiety-related behaviors, and social adjustment difficulties (Greenberg et al., 1992; Harmon-Jones et al., 1997; Lei and Zhang, 2003), then reduce or eliminate the generation of problem behaviors. The results indicate that individuals perceive higher SSS and self-esteem on their own will help to improve or reduce the level of adolescents’ problem behaviors.

The fact that SSS and self-esteem’ full mediation between psychological *suzhi* and problem behaviors supports Hypothesis 3, this result was similar to Hu and Zhang (2015), Liu et al. (2016), and Su and Zhang (2015). Possible explanations for this result are as follows. According to the relationship model between psychological *suzhi* and mental health, the state of mental health must rely on the stability and strong internal psychological *suzhi*’s support, psychological *suzhi* can be seen as the intrinsic foundation power of good mental health state to develop, and mental health can be regarded as the psychological *suzhi*’s explicit performance and behavior signs (Zhang et al., 2011; Zhang and Wang, 2012). Generally, higher psychological *suzhi* means higher cognitive quality and individuality (i.e., the ability to accurately understand and respond to objects and form reasonable beliefs), and higher adaptability (i.e., the ability to effectively control their behaviors; Hu and Zhang, 2015). In the present study, higher psychological *suzhi* may lead to better internal regulation to achieve higher SSS and self-esteem, and further affects adolescents’ problem behaviors. This indicates that it is an effective way to guarantee adolescents’ mental health (i.e., SSS, self-esteem, and problem behaviors) through cultivating adolescents’ sound psychological *suzhi* with the following mature methods and implementary strategies such as multimedia aided mode and implementation strategy, home-school cooperation mode and implementation strategy, and aesthetic nurturing mode and implementing strategy (Zhang, 2012; Zhang et al., 2014).

Moreover, we found that psychological *suzhi*’s indirect correlation with problem behaviors was significantly stronger via self-esteem than via SSS. Problem behaviors were more strongly correlated with self-esteem than SSS, and self-esteem and SSS were equally correlated with psychological *suzhi*. Possible explanations for this result may be as follows. For adolescence, they desire to get respect from their parents, teachers, and students, their own level of self-esteem also will rise and play an important role. For example, adolescents with high level of self-esteem tend to take the initiative to communicate with others, are easy to get the goodwill of others, and more likely to have good interpersonal relationships (Baumeister et al., 2003). Compared to self-esteem, SSS played a secondary role in adolescence, but played an important role in the upcoming contact with the society in college students (Chen et al., 2014). Therefore, psychological *suzhi*’s indirect effect on problem behaviors via self-esteem may exceed that of SSS.

The findings of the present study have important theoretical and practical implications. Whether it is psychological *suzhi*, or SSS and self-esteem, they all serve as significant predictors of problem behaviors and can thus be used as important tools to reduce the most frequent and harmful problem behaviors. Thus, when designing courses or interventions for reducing problem behaviors, researchers and educators should also focus on adolescents’ psychological *suzhi*, SSS, and self-esteem. Psychological *suzhi* affects adolescents’ problem behaviors via SSS and self-esteem, which extend the ways for interventions targeting problem behaviors in adolescents. Adolescence is a key stage for psychological *suzhi* and problem behaviors development. Teachers and educators should provide more related courses, activities to develop and improve adolescents’ psychological *suzhi*, SSS, and self-esteem, and then may reduce or eliminate problem behaviors among adolescents. According to the findings of the parallel mediating role of SSS and self-esteem between psychological *suzhi* and problem behaviors, first and most importantly, we should guide and facilitate adolescents’ psychological *suzhi* in their daily study life. Therefore, future research should test strategies for cultivating psychological *suzhi* in order to reduce or eliminate problem behaviors in adolescents.

Despite the theoretical and practical implications are discussed above, the present study has several possible limitations as follows. Firstly, although we used anonymous methods and used different self-report methods and formats to measure psychological *suzhi*, SSS, self-esteem, and problem behaviors, these methods may have improved the study’s internal validity and avoided or reduced common-method covariance. The one-sided self-report answers may have affected the level of the variables. To test the accuracy of the mediating model, future research should attempt to overcome this limitation by using other-report methods. Secondly, we used a cross-sectional design that cannot test possible causal relationships between the four variables. Therefore, future research should use an experimental or longitudinal design to test such possible relations. Thirdly, as the current study was conducted with a sample of students from three middle schools in China, whether the findings discussed above could be generalized to other middle schools’ students remains to be determined.

## Conclusion

This study expands the understanding of the mechanisms underlying *suzhi*’s effect on problem behaviors by the mediating roles of SSS and self-esteem, and its findings are novel and insightful, both theoretically and practically. This study not only clarifies that adolescents’ psychological *suzhi* negatively predicts their problem behaviors, but also supports the roles of their SSS and self-esteem as mediators in this relationship. Besides, self-esteem has a stronger indirect effect on problem behaviors than SSS does. In short, these findings suggest that SSS and self-esteem underlie psychological *suzhi*’s effect on adolescents’ problem behaviors. To this end, the current study offers an important foundation for future work.

## Ethics Statement

The Research Ethics Committee of Southwest University approved the study. Prior to testing, we obtained both written and informed consent from all participants and their parents. Willing participants subsequently provided spoken consent.

## Author Contributions

On the basis of reading the relevant literature, GL put forward the research questions and the solution of this research, and the whole research process is responsible for the research plan of this research, for example, to determine the research object, clear research methods, contact the subjects to test, data entry, and so on, and finally to collect the data back analysis to verify the assumptions made by this study, and is responsible for the study of the full text of the writing work. At the same time, according to the teachers’ and cooperative researchers’ recommendations and comments on the draft of the paper have been amended to formally form the final submission manuscript. DZ was mainly responsible for the supervision and guidance of the entire research process, timely correct the wrong ideas and direction, and the smooth completion of the entire study plays an important role. DZ was also mainly responsible for modifying and providing guidance to this paper. YP was mainly responsible for modifying and providing guidance to this paper, played an important role in the successful writing of the paper. YM was mainly responsible for modifying and providing guidance to this paper, also played an important role. XL was mainly responsible for language polish.

## Conflict of Interest Statement

The authors declare that the research was conducted in the absence of any commercial or financial relationships that could be construed as a potential conflict of interest. The reviewer KG and handling Editor declared their shared affiliation.
